# Convergent Strabismus

**Published:** 1872-01

**Authors:** C. A. Simpson

**Affiliations:** Atlanta, Georgia


### CONVERGENT STRABISMUS.

BY DR. C. A. SIMPSON, ATLANTA, GEORGIA.

I have had opportunities of seeing a good many operations for strabismus, in the eye and ear hospitals of this city, in which there were, almost without exception, excellent results. The operation is usually performed in the following manner: The patient is almost always put under the influence of ether or chloroform—ether is preferred by most of the surgeons here. The eye-lids are held open by a spring speculum, and (supposing the operation to be on the internal rectus for convergent strabismus) an assistant seizes the ocular conjunctiva with the forceps on the outer side of the globe, and rotates it outward. The surgeon then grasps with the forceps the conjunctiva near the inner edge of the cornea, just over the internal rectus, and between the middle and lower edge—raises it in a fold, and with a pair of blunt-pointed strabismus scissors, curved on the flat, cuts sharply through the conjunctiva down to the sclerotic, opening up the conjunctiva more or less extensively, according to the effect desired to be produced by the operation. A blunt-pointed strabismus hook is then inserted through the opening in the conjunctiva, passed beneath the lower edge of the tendon of the internal rectus, and raising it sufficiently to pass one prong of the scissors beneath the tendon of the muscle, it is snipped through as closely as possible to its attachment to the globe. The hook must then be passed up and down beneath the conjunctiva, and any remaining fibres raised and divided in the same manner. When every fibre has been divided, the wound in the conjunctiva may or may not be brought together by a suture, according to the result wished to be produced.

The after-treatment is very simple: cold water to be applied for a day or two, the patient kept quiet, and the eye to be protected from too much light.

Almost all cases of squint are either convergent or divergent. A great proportion of these are convergent. Convergent strabismus is in most cases due to hypermetropia, which is often hereditary, and observed in several members of the same family, thus giving ground for the popular belief that squint may be caused by imitation. Hypermetropia is that condition in which the antero-posterior diameter of the eye is shorter than the normal, and rays of light coming from a distant object (parallel rays), instead of being brought to a focus on the retina, are brought to a focus behind it. To cause these rays to be focused on the retina, the hypermetropic eye requires a convex glass; or, in slight degrees of hypermetropia, this may be accomplished by exerting the accommodation. The nearer an object is brought to the eye, the more the rays from it diverge, and the more is the accommodation exerted to cause them to converge sufficiently to be focused on the retina.

In hypermetropia, if an object is brought very near, one eye turns inward, because the power of accommodation increases with the increased degree of convergence of the visual lines. This may be shown in the following way: If a weak concave glass be placed before an emmetropic eye, (in which the antero-posterior diameter is of normal length), the rays are made to diverge so much that they come to a focus behind the retina, and the eye becomes in effect hypermetropic. The eye can, by an effort of its accommodating power, if the glass be not too strong, focus the rays on the retina. If a glass be placed before the eye, so strong that the rays cannot be brought to a focus on the retina by a mere exertion of the accommodation, one eye will be seen to squint inward.

Convergent strabismus is sometimes also due to impaired vision of one eye from opacity of the cornea, lens, or disease of some of the deeper structures of the eye. There is a great difference in the distinctness of the images formed upon the retina in the two eyes, which is very annoying. In order to diminish this, the unsound eye squints inward, so that the rays may impinge upon a less sensitive portion, and so weak an image be formed that it may be mentally suppressed. Sometimes the eye squints in some other direction. It is drawn in the direction of the strongest muscle.

Convergent strabismus may be a secondary affection, and caused by the external rectus being paralyzed, divided, or otherwise injured. This is called secondary squint, and may also sometimes be caused by too extensive an operation for strabismus, or dividing the muscle instead of the tendon.

Myopia (short-sightedness) may sometimes be the cause of a convergent squint. This occurs in persons who are constantly employed at very near work, and in whom the myopia is of a very moderate degree. It is due to the eyes being kept in such a constant state of convergence, by this near work, that the internal recti become so contracted that, when the person looks at a distant object, the external recti are too weak to overcome this contraction, and a squint is the result. This is one of the ill effects of myopic persons doing very close work.

Convergent strabismus is usually very evident to all around, from the deformity it causes, and is popularly known as squint or crossed eyes. Something more is required of the physician than such a loose diagnosis as this.

In convergent strabismus, (I leave out strabismus divergens, or squint outward, strabismus sursum vergens, or squint upward, strabismus deorsum vergens, or squint downward), if the patient be directed to look at some object—say a pencil held up before him—one eye will be observed to be fixed on this object, while the other will be turned inward at a certain angle. In order to determine which is the squinting eye, if you direct the patient to look at the object, and cover each eye alternately with your hand, it will be perceived that one of the eyes, when the other is covered, will not change its position to fix the object; but if the hand be placed over this eye, it will be seen that the one which is now left uncovered turns a little outward in order to fix the object. Sometimes this movement outward is so slight as not to be observed; and we must test the patient with prisms, to see if there be diplopia, or double sight, as this shows that the visual lines of the two eyes are not at the same angle, and the rays from the object do not fall upon corresponding parts of the retina.

In a squint which is very evident, if the sound eye be covered with a piece of ground glass while the patient is looking at an object, the squinting eye will move outward to fix the object, and the sound eye can be seen through the glass to turn inward, and now becomes the squinting eye. This is called the secondary deviation. In this secondary deviation, the movement inward of the sound eye corresponds exactly with the outward movement of the squinting eye, and it is called concomitant squint. If the secondary deviation is greater than the primary (deviation of the squinting eye), it is not a case of concomitant squint, but there is paralysis of the external rectus muscle, which may be demonstrated in the following way: If the external rectus be paralyzed, (say of left eye), and you hold an object before the patient, and move it gradually to the left, directing him to follow it with his eyes without turning the head, when the object is carried far to the left, the left eye will not be able to follow it as far as the right, owing to the inability of the paralyzed external rectus to draw it out any further, and a consequent squint will be the result. If now the object be held in front of the eyes, and the right (or sound eye) be covered with a piece of ground glass, so that its movements may be seen without its being able to distinguish objects, the left eye will immediately make a movement outward—say of one and a half lines; the inward or secondary movement of the right will be considerably more—two and a half or three lines.

The degree of strabismus should be determined for both near and distant objects, as the squint is almost always much greater when looking at objects very near than when looking at some distant object. It should be determined, too, whether the superior or inferior rectus is concerned in the squint. If, when the sound eye is covered, and the squinting eye fixes the object, the deviating sound eye is of the same height as the squinting eye, it is only the internal rectus which is at fault, and it will be necessary to operate only on this muscle; but if there be a difference in the height of the two eyes, one of the other muscles is also at fault. In some cases, this difference in height may be due to the upper fibres of the internal rectus being much stronger than the rest of the muscles, and consequently drawing the eye upward as well as inward.

The accommodating movements of the squinting eye should also be tested. If an object be held in front of the patient, a foot or two from his nose, and he be directed to look directly at it, as the object is carried nearer and nearer the nose, the sound eye converges more and more, while the squinting eye will act in one of the following ways:

1. It may retain its original position, sustaining only a few oscillating, irregular lateral movements.

2. It may remain completely stationary, and when the object is at a certain distance from the nose, the visual lines of the squinting eye and sound eye will be fixed on the object, and there will no longer be an appearance of a squint. If, however, the object be carried still closer, there will be a divergent squint; for while the sound eye converges still more, the other retains its position, and now deviates (passively) outward.

3. It retains its position when the object is brought up to a certain point, and then, as the healthy eye moves inward to follow the object, it makes an associated movement outward.

4. It deviates suddenly and spasmodically inward when the object is brought very close.

In some cases of convergent strabismus, one eye alone squints, (monolateral strabismus), while in others either eye may squint—sometimes one, and sometimes the other—alternating strabismus.

In monolateral strabismus, if the healthy eye be covered while the patient is looking at an object held in front of the eyes, the squinting eye will be noticed to turn outward to fix the object. If, now, the hand be removed from the healthy eye, it will fix the object, and the squinting eye will turn inward. In alternating strabismus, if the above test be applied, each eye will remain fixed on the object when the other is uncovered, the uncovered eye retaining the squint—thus showing that one and sometimes the other eye squints.

In alternating strabismus, the vision of the two eyes is usually the same, while in monolateral strabismus, the sight of the squinting eye is usually much less than that of the sound eye, from the image in the squinting eye being mentally suppressed to avoid distressing diplopia.

It will be thus seen that a monolateral is more serious than an alternating strabismus, as in one the sight of the squinting eye may be seriously impaired from non-use, while in the other case there is no such danger as this, as both eyes are used alike.

Now as to the prognosis and treatment of strabismus convergens. It is more frequently first noticed in children when they first begin to learn their letters, and is usually caused by hypermetropia. The squint may at first be periodic, and only noticed when the child looks at near objects; but, if not treated, it soon becomes permanent. The periodic squint may sometimes be cured by a use of the proper convex glasses to correct the hypermetropia.

If the squint can only be cured by an operation—which, for some reason, has to be postponed—the squinting eye should be frequently exercised by covering the other; for on account of the confusing double images, that of the squinting eye is mentally suppressed; and if this continues for a long time, the vision of this eye becomes very much reduced.

Sometimes, when the strabismus is slight, it may be corrected by a systematic exercise of the contracted muscle by means of prisms. This is a long and very tedious treatment. It is only indicated when the vision of the squinting eye is not too much deteriorated—when there is binocular vision, and when the double images are so near that the patient can unite them by an effort.

In most cases, a squint can be cured only by an operation. The operation, in some cases, is for the purpose of both straightening the eye and improving the vision; in others, merely for straightening the eye, without the expectation of improving the sight; and in other cases, where the squint is scarcely, if at all perceptible, the operation is for the purpose of fusing the double images, which are very annoying.

NEW YORK CITY, December 26th, 1871.

